# Regulation of pyruvate dehydrogenase complex related to lactate switch in CHO cells

**DOI:** 10.1002/elsc.202000037

**Published:** 2020-09-28

**Authors:** Johannes Möller, Krathika Bhat, Lotta Guhl, Ralf Pörtner, Uwe Jandt, An‐Ping Zeng

**Affiliations:** ^1^ Bioprocess and Biosystems Engineering Hamburg University of Technology Hamburg Germany

**Keywords:** dynamic enzyme regulation, lactate switch, PDC phosphorylation, Warburg effect

## Abstract

The metabolism of Chinese hamster ovary (CHO) cell lines is typically characterized by high rates of aerobic glycolysis with increased lactate formation, known as the ”Warburg” effect. Although this metabolic state can switch to lactate consumption, the involved regulations of the central metabolism have only been partially studied so far. An important reaction transferring the lactate precursor, pyruvate, into the tricarboxylic acid cycle is the decarboxylation reaction catalyzed by the pyruvate dehydrogenase enzyme complex (PDC). Among other mechanisms, PDC is mainly regulated by phosphorylation–dephosphorylation at the three sites Ser232, Ser293, and Ser300. In this work, the PDC phosphorylation in antibody‐producing CHO DP‐12 cell culture is investigated during the lactate switch. Batch cultivations were carried out with frequent sampling (every 6 h) during the transition from lactate formation to lactate uptake, and the PDC phosphorylation levels were quantified using a novel indirect flow cytometry protocol. Contrary to the expected activation of PDC (i.e., reduced PDC phosphorylation) during lactate consumption, Ser293 and Ser300 phosphorylation levels were 33% higher compared to the phase of glucose excess. At the same time, the relative phosphorylation level of Ser232 increased steadily throughout the cultivation (66% increase overall). The intracellular pyruvate was found to accumulate only during the period of high lactate production, while acetyl‐CoA showed nearly no accumulation. These results indicate a deactivation of PDC and reduced oxidative metabolism during lactate switch even though the cells undergo a metabolic transition to lactate‐based cell growth and metabolism. Overall, this study provides a unique view on the regulation of PDC during the lactate switch, which contributes to an improved understanding of PDC and its interaction with the bioprocess.

## 1 INTRODUCTION

The majority (approx. 80%) of recombinant protein therapeutics are currently being produced using mammalian cell lines, such as Chinese hamster ovary (CHO) cells [1, 2, 3]. Even though CHO cells have been used for over two decades in the biopharmaceutical industry, a major drawback is the ”Warburg” effect characterized by high glucose uptake and lactate production rates during exponential cell growth [4, 5]. Depending on the cell line, lactate concentrations of 20–60 $\mathrm{mmol}\;L^{-1}$ have been found to be inhibitory to cell growth and productivity [6, 7, 8]. Thus, many process modifications and genetic engineering approaches have been used to reduce lactate formation and improve the metabolic efficiency of the cells [9, 10, 11]. An approach of particular interest is the triggering of the cell metabolism to take up lactate and utilize it as a secondary substrate (i.e., lactate switch) [12, 13]. This shift to lactate consumption is described to be beneficial due to an increased process performance with prolonged culture viability [12, 14, 15]. Novel cultivation concepts have tried to control the metabolic switch to lactate uptake [16]. Exemplary, [17] and [18] used pH shifts to induce co‐consumption of glucose and lactate [17, 18].

The major reaction diverting the glycolysis‐derived pyruvate flux away from lactate formation and allowing its entry into the tricarboxylic acid cycle (TCA) cycle is the decarboxylation reaction catalyzed by the pyruvate dehydrogenase enzyme complex (PDC) [19]. PDC is a key enzyme complex in mammalian cell metabolism, and it is tightly regulated by a phosphorylation–dephosphorylation mechanism at the three Serine (Ser) residues Ser232, Ser293, and Ser300 of the PDCE1α subunit [20, 21]. The regulation of PDC gains recognition due to its importance in cell metabolism and its highly dynamic nature [22, 23]. Furthermore, the function of PDC as a macromolecular machine is focused on to design artificial multistep reactions [24, 25, 26, 27]. It is targeted to synthetically modify PDC to accept different substrates or to enable other reactions [25]. However, a structural orientation of the PDC subunits and their interaction is not fully understood, and mostly molecular dynamic studies are currently performed to understand them before synthetic modifications can be targeted [26].

**PRACTICAL APPLICATIONS**

Changes in cell metabolism in mammalian producer cell lines are characterized by ineffective glucose metabolism with high lactate formation. Furthermore, cells may utilize lactate as a secondary substrate and shift their metabolism (lactate shift). Nonetheless, the role of the pyruvate dehydrogenase complex (PDC) regulation during the metabolic shift from lactate formation to lactate uptake has not yet been sufficiently investigated. With this study, the interaction of oxidative metabolism with the PDC regulation by phosphorylation–dephosphorylation was descriptively studied for the first time during the lactate shift. Contrary to the general assumption of increased PDC activity (i.e., reduced phosphorylation) during lactate uptake, the results indicate that PDC is deactivated (i.e., increased phosphorylation) during lactate consumption compared to the glucose excess phase. These results contribute toward the recent efforts in understanding this metabolic switch, as it is important for both mammalian cell culture and human diseases.

Based on the current understanding of PDC regulations, an increased phosphorylation of PDC (i.e., PDC deactivation) could be expected during the exponential growth phase with high lactate formation [13]. During lactate uptake, a reduced PDC phosphorylation (i.e., PDC activation) could be presumed to allow pyruvate to enter the TCA cycle, since lactate consumption has been associated with increased oxidative metabolism [12, 28, 29]. However, it is still not clear if changes in the PDC regulation are characteristic hallmarks for lactate switch in mammalian cell culture [13].

In this work, the regulation of PDC by phosphorylation and dephosphorylation was experimentally investigated during the transition from lactate production to lactate consumption in antibody‐producing CHO DP‐12 cultures. The relative phosphorylation status of the regulating sites Ser232, Ser293, and Ser300 on PDC E1α was determined in batch cultures with frequent sampling (every 6 h). Furthermore, cell death effects (i.e., apoptosis, extracellular lactate dehydrogenase (LDH) activity), the intracellular concentrations of pyruvate and acetyl‐CoA, and the changes of extracellular components (glucose, lactate, free amino acids, ammonium, antibody) were analyzed during the lactate switch.

### 1.1 Regulation of the pyruvate dehydrogenase complex

**FIGURE 1:**
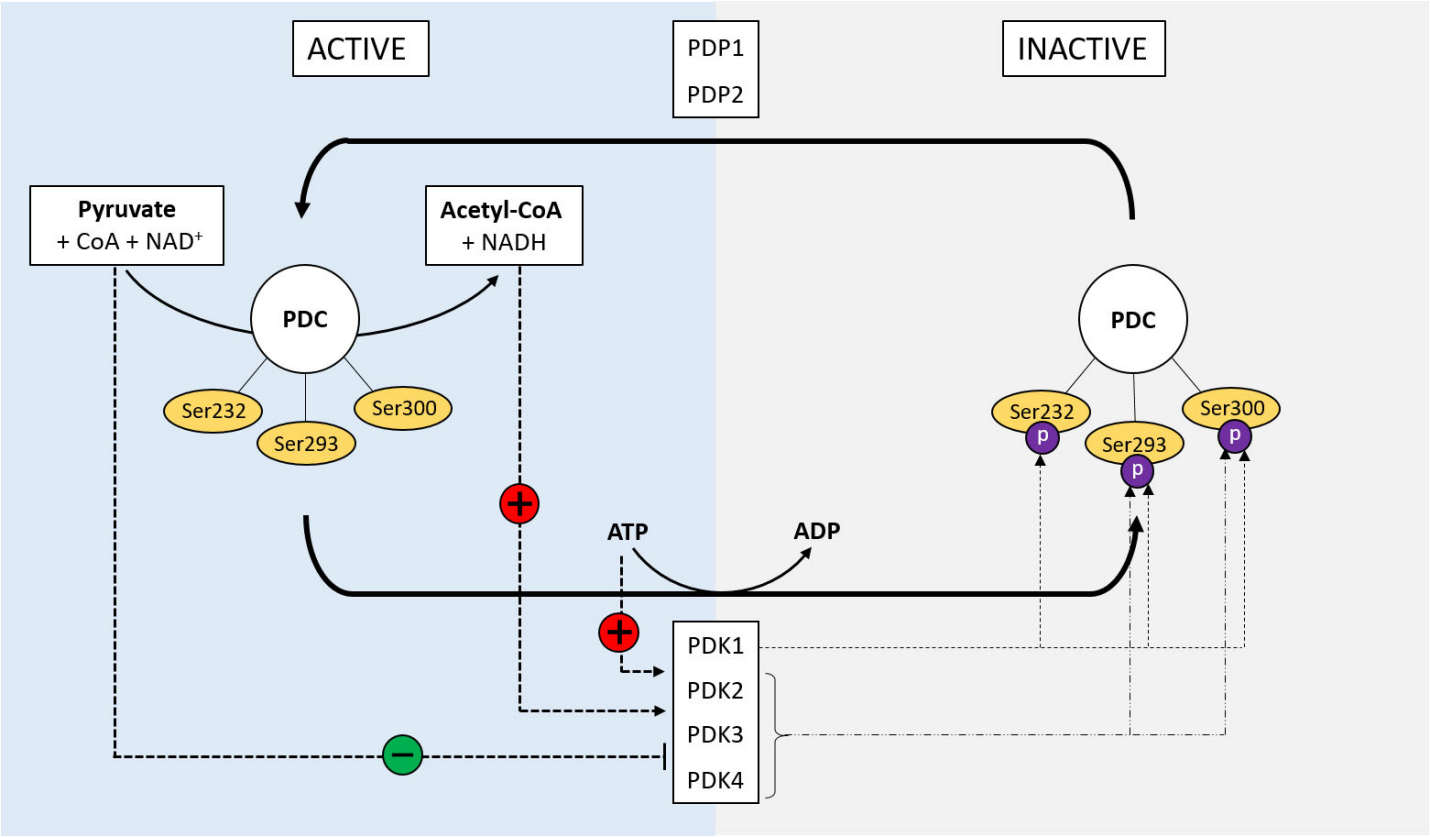
Regulatory mechanisms of PDC by phosphorylation and dephosphorylation. + = stimulation; ‐ = releasing; green color = PDC activation, red color = PDC deactivation

Each of the Ser residues (Ser232, Ser293, and Ser300, respectively, in hamster [30]) can be modified by two groups of enzymes, namely pyruvate dehydrogenase kinase (PDK) and pyruvate dehydrogenase phosphate phosphatase (PDP). PDC is deactivated by phosphorylation of the Ser PDCE1α sites through four PDKs, which exhibit differences with respect to site‐specificity and phosphorylation rate. Two different PDP isoforms (PDP1 and PDP2, respectively) activate PDC by dephosphorylation of the phosphorylated Ser PDCE1α sites (pSer‐ phosphorylated Ser), which have the same degree of activity towards the regulating sites [31]. The mechanisms regulating the activity of PDC through the stimulation and inhibition of PDK's and PDP's, and the phosphorylation–dephosphorylation reaction of the pSer PDCE1α are schematically shown in Figure 1. Short‐term changes in PDC activity are influenced by changes in metabolites (left part in Figure 1). The products of the reaction catalyzed by PDC, namely NADH and acetyl‐CoA, activate the PDK's and their accumulation results in a reduction of PDC activity [32]. On the other hand, the increased availability of intramitochondrial pyruvate inhibits the PDK's and will, therefore, activate PDC [33, 34]. A drop in ATP level stimulates PDP's, which also results in increased PDC activity [34]. It has been found in experiments with purified enzymes that phosphorylation at Ser293 is the most rapid one (short‐term regulation), whereas Ser300 is phosphorylated 4.6‐times slower than Ser293, and Ser232 phosphorylation is 16‐fold slower [31]. PDK1 phosphorylates all Ser and PDK2, PDK3, and PDK4 can only phosphorylate Ser293 and Ser300 [35]. Although in vitro studies indicate that PDC phosphorylation at any one site is sufficient to deactivate the whole complex, the mechanism of deactivation shows site‐specific differences [31]. Exemplary, [35] identified that the phosphorylation of Ser293 affects the interaction of E1 with the lipoyl domains of E2, while the phosphorylation of Ser232 is associated with changes in the TPP‐binding regions [35].

Further insights into the highly dynamic nature of PDC as a multienzyme complex could be achieved using mathematical modeling approaches. [36] used molecular dynamic simulations to test the structural consequences of the phosphorylation on PDCE1α. They found that phosphorylations of Ser293 and Ser300 change the PDCE1α catalytic site [36]. As a different approach, [22] developed an in vitro and in vivo kinetic model of the PDC reaction network and [23] used the same model to study the influence of pyruvate concentration and suggested that the PDC reaction rate is a non‐linear function of its substrate. Moreover, multiple steady states were characterized, and a more efficient state was found for a rather low initial pyruvate concentration [23].

## 2 MATERIAL AND METHODS

### 2.1 Cell line and culture medium

Suspension‐adapted CHO DP‐12 cells (clone #1934, ATCC CRL‐12445, kindly provided by Prof. T. Noll, Bielefeld University, Germany) producing an interleukin‐8 antibody were used in all experiments conducted in this work. The cell line was cultivated in chemically‐defined, animal component‐free TC‐42 medium, available with or without glucose (Xell AG, Germany). The medium was supplemented with variable concentrations of glucose, 6 mmol $L^{-1}$ of glutamine and 200 nmol $L^{-1}$ of methotrexate (all Sigma‐Aldrich, Germany).

### 2.2 Preculture

The cells were precultured in medium containing $42\;\mathrm{mmol}\;L^{-1}$ glucose, 6 mmol $L^{-1}$ of glutamine and 200 nmol $L^{-1}$ methotrexate (Xell AG, see Subsection 2.1). A single‐use Erlenmeyer baffled flask (40 mL working volume, Corning, Netherlands) with pre‐warmed medium was inoculated with 1 mL of freshly thawed cryoculture ($10^{7}\;\mathrm{cells}\;{\mathrm{mL}}^{-1}$). The conditions inside the shaker (LT‐X, Kuhner AG, Switzerland) were $37^{\circ }C$ temperature, 5% ${\mathrm{CO}}_{2}$ and 85% humidity, with a shaking frequency of 200 rpm (25 mm shaking diameter).

### 2.3 PDC phosphorylation during lactate switch

Two batch cultivations were performed in the benchtop bioreactor Vario 1000 (Medorex, Germany). One cultivation targeted the PDC phosphorylation and is discussed in the main manuscript, whereas the second cultivation was performed to validate the intracellular pyruvate fluxes and is shown in Supporting Information Subsection 2.3. In both cultivations, the initial working volume was 200 mL fresh medium containing $20\;\mathrm{mmol}\;L^{-1}$ glucose, $6\;\mathrm{mmol}\;L^{-1}$ glutamine and 200 nmol $L^{-1}$ methotrexate (same as in Subsection 2.1). The initial cell density was $0.3\cdot 10^{6}\;\mathrm{cells}\;{\mathrm{mL}}^{-1}$. Air was supplied to the headspace at a constant rate of $10\;\mathrm{ml}\;\min^{-1}$ throughout the culture duration. The dissolved oxygen level was maintained above 40% via sparging of pure oxygen into the culture medium if required and the pH was maintained at 7.1 (sparging with ${\text{CO}}_{2}$ or addition of $0.5\mathrm{mol}\;L^{-1}\;{\mathrm{Na}}_{2}{\mathrm{CO}}_{3}$). The temperature was maintained at $37^{\circ }C$ and the impeller speed was set to 400 rpm. The sampling interval was 24 h from t=0−72h and then reduced to 6 h until the end of the cultivation. The offgas composition was measured using an analyzer (BlueVary, BlueSens, Germany).

### 2.4 Cell concentration, diameter, and viability

Total cell concentration and cell size distribution were measured with the Z2 particle counter (Z2, Beckman Coulter, USA) as explained in [37]. The cell suspension was centrifuged (300g, 3min), the supernatant was frozen ($-20^{\circ }C$) for metabolite analysis, and the cells were suspended in phosphate‐buffered saline (PBS, $4^{\circ }C$). The viability was determined with DNA staining using the DAPI (4',6‐diamidino‐2‐phenylindole) method. Therefore, cells were suspended in $4^{\circ }C$ PBS with $1\;\mu g\;{\mathrm{mL}}^{-1}$ DAPI and immediately measured with flow cytometry (Cytoflex, Beckman Coulter). The 405nm laser and 450/50nm filter signal were used. Debris and doublets were excluded with side scatter‐A vs. forward scatter (FSC)‐A and FSC‐H vs. FSC‐A gating, and non‐stained cells were gated as viable (20 000 recorded events, CytExpert Software, Beckman Coulter).

### 2.5 Quantification of apoptotic cells

The proportion of healthy and apoptotic cells in the culture was quantified by annexin V and propidium iodide (both BD Biosciences, USA) staining. $10^{5}$ cells in 100μL of 1x binding buffer were labeled using 5μL of V450 annexin V followed by 5μL of propidium iodide solution ($50\;\mu g\;{\mathrm{mL}}^{-1}$), and incubated at room temperature ($\approx 20^{\circ }C$) for 15 min in the dark. Then, the cells were analyzed using flow cytometry (Cytoflex, Beckman Coulter). Debris and doublets were excluded and the V450 annexin V fluorescence was detected using the 405nm laser and 450/50nm filter signal. The 488nm laser and 690/50nm filter signal were used for the propidium iodide signal. 30 000 events were recorded (CytExpert). Please see Supporting Information Subsection 1.1 for more information about gating.

### 2.6 LDH activity

The LDH activity in the culture medium was quantified to determine the degree of cell lysis using a colorimetric assay kit (#ab102526, Abcam, USA). The assay was carried out as per the manufacturer's protocol.

### 2.7 Metabolite analysis

#### 2.7.1 Extracellular metabolites

Concentrations of glucose ($c_{\mathrm{Glc}}$), glutamine ($c_{\mathrm{Gln}}$), and lactate ($c_{\mathrm{Lac}}$) were quantified with the YSI 2900D (Yellow Springs Instruments, USA) biochemistry analyzer. The ammonium concentration ($c_{\mathrm{Amm}}$) was determined with an enzymatic test kit (AK00091, nzytech, Portugal). The antibody titer ($c_{\mathrm{Ab}}$) was determined using bio‐layer interferometry (Octet RED, Pall ForteBio, USA) with protein A biosensors (Pall ForteBio). Amino acid concentrations were determined from pre‐column derivatized supernatant (ortho‐phthalaldehyde method) by reversed‐phase chromatography using a Poroshell HPH‐C18 column (125 x 4.6 mm, pore size 0.5μm, Agilent Technologies, USA) with fluorometric detection [38].

#### 2.7.2 Intracellular metabolites

Intracellular pyruvate ($c_{\mathrm{Pyr}}$) and acetyl‐CoA ($c_{\mathrm{AcCoA}}$) concentrations were quantified with assay kits (#ab65342 and #ab87546, Abcam, USA). The cells harvested from the bioreactor ($\approx \;2\cdot 10^{6}$ cells) were washed with PBS (1000g, 3 min) and the cell pellets were snap‐frozen and stored in liquid nitrogen (1.8 ml cryovials, Thermo Scientific, Germany). For the quantification of intracellular metabolites, the snap‐frozen cell pellets were resuspended in assay buffer (containing surfactant, $4^{\circ }C$) and incubated for 5 min on ice with intermediate vortexing. Then, the suspension was centrifuged at 21100g for 5 min at $4^{\circ }C$ to obtain debris‐free lysate (i.e., supernatant). Deproteinization was carried out using a 10 kDa spin filter (Amicon Ultra‐0.5 mL Centrifugal Filter, Merck, Germany) with centrifugation at 14000g for 10 min at $4^{\circ }C$. Subsequently, the assays were carried out according to the manufacturer's protocol.

#### 2.7.3 Metabolic rates

The cell‐specific uptake/formation rates were calculated using the measured metabolite concentrations ($c_{j,i}$) at two consecutive time points ($t_{i},\;t_{i+1}$) and the measured viable cell concentration ($X_{v}$) as follows:

(1) $$q_{j}\left(t_{i},t_{i+1}\right)=\frac{c_{j,i}-c_{j,i+1}}{t_{i+1}-t_{i}}\cdot \frac{2}{X_{v,i+1}+X_{v,i}}$$

#### 2.7.4 Oxygen uptake rate

The cell‐specific oxygen uptake rate ($q_{\mathrm{OUR}}$) was calculated using data averaged over t=12h intervals. Therefore, the oxygen input flow rate and the oxygen offgas flow rate were averaged over t=12h intervals and the difference between the average values were considered to be the total oxygen consumed (${\mathrm{OUR}}_{\mathrm{avg}}$) in the t=12h interval. $q_{\mathrm{OUR}}$ was calculated based on the oxygen density at $37^{\circ }C$ ($\rho_{O_{2}}=1.24\;g\;L^{-1}$), the molecular weight of oxygen ($M_{O_{2}}$), and the liquid bioreator volume ($V_{l}$):

(2) $$q_{\mathrm{OUR}}=\frac{{\mathrm{OUR}}_{\mathrm{avg}}\cdot \rho_{O_{2}}}{M_{O_{2}}\cdot X_{v,\mathrm{avg}}\cdot V_{l}}.$$

### 2.8 Analysis of PDC phosphorylation

The phosphorylation of the PDCE1α sites was quantified with indirect flow cytometry through the labeling of the phosphorylated Ser residues pSer232, pSer293, and pSer300. The detailed assay procedure and calculations using the flow cytometry data are provided in the Supporting Information Subsection 1.2. Briefly, $4.5\;\cdot \;10^{6}$ cells were fixed during routine sampling using 4% paraformaldehyde solution (Histofix, Roth, Germany) for 20 min and washed with PBS [39]. The final density of fixed cells was determined by flow cytometry (Cytoflex, Beckman Coulter) and adjusted to $0.5\cdot 10^{6}\;\mathrm{cells}\;{\mathrm{mL}}^{-1}$ using PBS. The assay was carried out in 96‐well flat‐bottom black plates (Thermo Fisher Scientific, USA) with 200μL of the fixed cell suspension per well. Subsequently, the cells were treated with different buffers (see Table 1) in a series of steps with intermediate washing. The washing step involved centrifuging the plate (Hettich Universal 320 R, Hettich, Germany) at 400g for 15min followed by automated replacing 150μL of the supernatant with washing buffer (Biomek 4000, Beckman Coulter). These steps were repeated twice (centrifugation time reduced to 10min) and they together constitute the washing sequence.

**TABLE 1:** Buffers for indirect flow cytometry assay to quantify PDC E1α phosphorylation. Between all steps, intermediate washing was performed

	**Buffer composition [% (v/v)]**
**Permeabilization**	2% Tween 20 (Sigma‐Aldrich)
	98% PBS (Sigma‐Aldrich)
**Blocking**	10% blocking solution (Abcam, In‐Cell ELISA support pack)
	10% fetal calf serum (FCS) (Biochrom, Germany)
	80% 0.33 $\mathrm{mol}\;L^{-1}$ Glycine (Sigma‐Aldrich) in washing buffer
**Staining**	10% blocking solution (Abcam, In‐Cell ELISA support pack)
	10% FCS
	80% PBS
**Washing**	0.25% Tween 20 (400x, Abcam, In‐Cell ELISA support pack)
	99.75% PBS

First, the cells were permeabilized using the permeabilization buffer for 20 min. Then, the cells were incubated in a blocking buffer for 2 h after which staining with antibodies was performed. The antibodies used are listed in Table 2 along with their final concentration in each well. More details about the Anti‐PDC E1α‐related antibodies can be found in [40]. The antibodies were first added to the staining buffer, which was then added to the wells. All wells were stained with the primary antibody specific to the PDC E1α subunit. Then, the cells were divided into four groups: one group without anti‐pSer antibodies (i.e., quantification of total PDC E1α), three groups stained with anti‐pSer antibodies, separated as one group for each pSer residue (pSer232, pSer293, pSer300, respectively). The incubation with primary antibodies was carried out for 16 h and with secondary antibodies for 20 min, and the cells were washed and analyzed by flow cytometry (Cytoflex, Beckman Coulter).

**TABLE 2:** Reagents for indirect flow cytometry assay to quantify PDC E1α phosphorylation

**Reagent**	**Concentration**
Anti‐PDC E1α pSer232 (polyclonal‐rabbit, Merck, Germany)	$1\;\mu g\;{\mathrm{mL}}^{-1}$
Anti‐PDC E1α pSer293 (polyclonal‐rabbit, Merck)	$1\;\mu g\;{\mathrm{mL}}^{-1}$
Anti‐PDC E1α pSer300 (polyclonal‐rabbit, Merck)	$1\;\mu g\;{\mathrm{mL}}^{-1}$
Anti‐PDC E1α (monoclonal‐mouse, G‐biosciences, Alexa‐fluor 488)	$1.25\;\mu g\;{\mathrm{mL}}^{-1}$
Anti‐rabbit (polyclonal‐goat, Abcam, Alexa‐fluor 405)	$0.25\;\mu g\;{\mathrm{mL}}^{-1}$

From the flow cytometer data, the median of the individually gated positive intensities (E1α positive‐$\tilde{I_{E1\alpha }}$ and pSerE1α positive‐$\tilde{I_{\mathrm{pSer},E1\alpha }}$, respectively) were determined and the relative phosphorylation P was quantified:

(3) $$P=\frac{\tilde{I_{\mathrm{pSer},E1\alpha }}}{\tilde{I_{E1\alpha }}}.$$

Considering fluctuations in biological samples and data measurements, P was standardized on the non‐primary labeled intensity $P_{0}$ (i.e., no pSer antibody) measurements, and pSer (relative) = $\frac{P}{P_{0}}$ was used for data analysis.

## 3 RESULTS AND DISCUSSION

**FIGURE 2:**
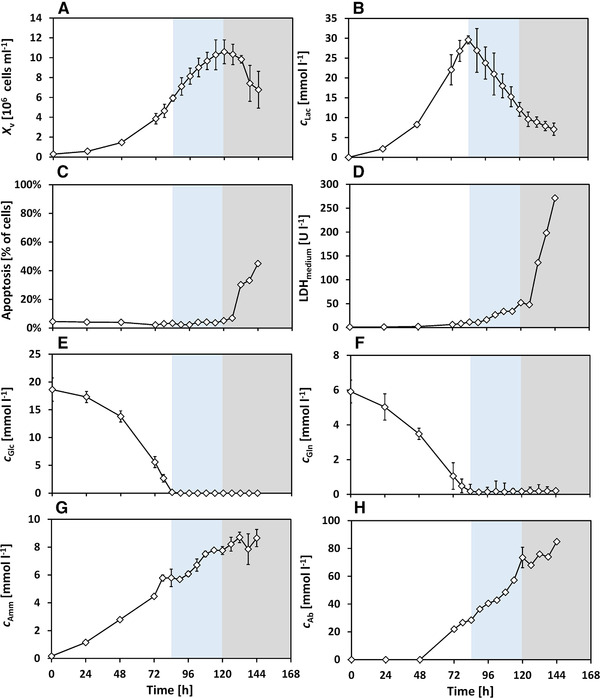
Mean experimental results from the batch cultivation with metabolic shift from lactate formation to lactate consumption. Phases indicated: lactate formation phase (white), lactate consumption phase (blue) and death phase (grey). Error bars of $c_{\mathrm{Amm}}$, and $c_{\mathrm{Ab}}$ show one‐fold standard deviation of three technical measurements; error bars of $X_{v}$, $c_{\mathrm{Glc}}$, $c_{\mathrm{Lac}}$, $c_{\mathrm{Gln}}$ show 10‐fold standard deviation for better visibility, calculated on three technical measurements

**FIGURE 3:**
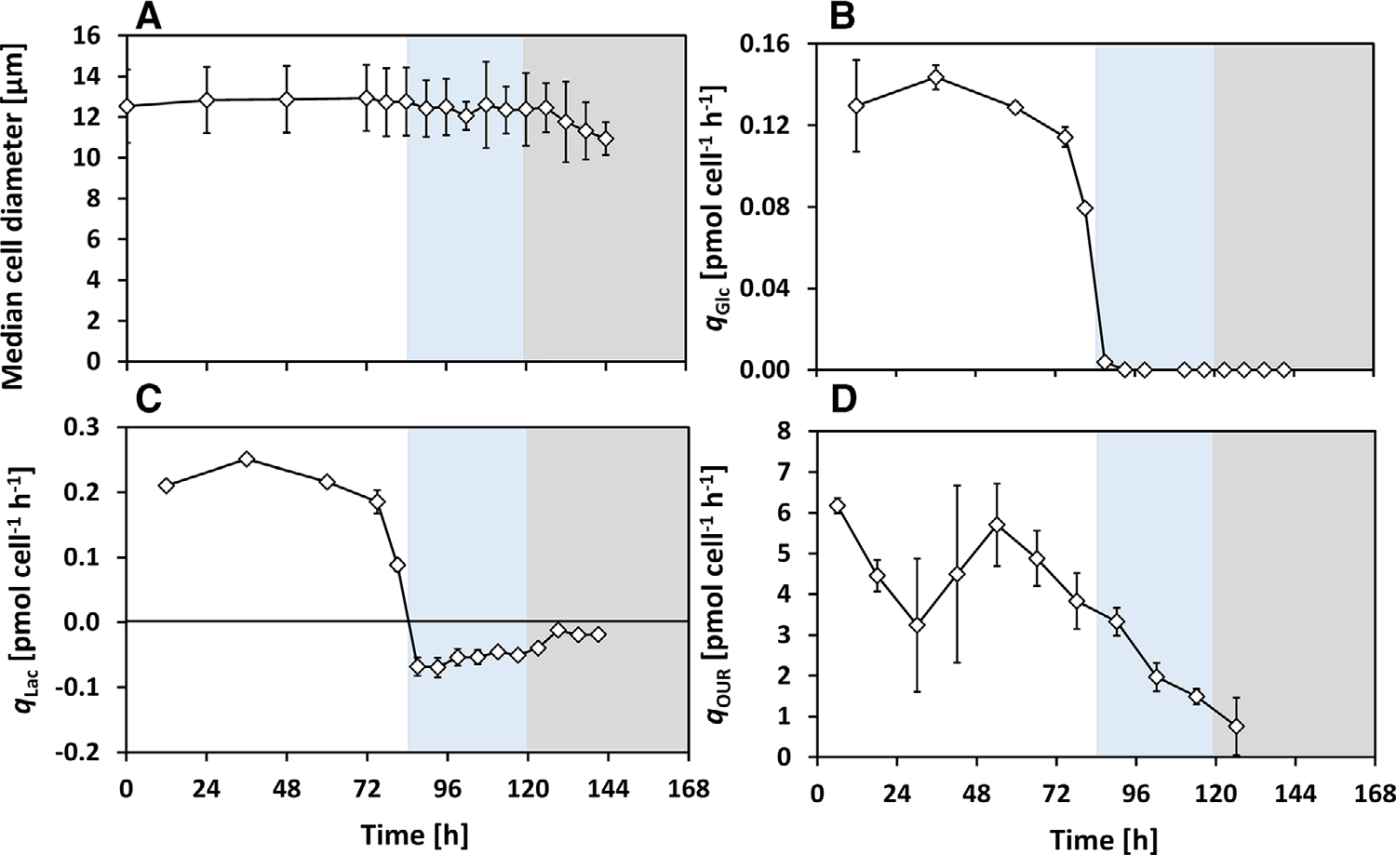
Cell diameter changes and cell‐specific rates during the batch cultivation with metabolic shift to lactate consumption. Phases indicated: lactate formation phase (white), lactate consumption phase (blue) and death phase (grey). Error bars show 10‐fold standard deviation (A), or one‐fold standard deviation (B–D) of three technical measurements

**FIGURE 4:**
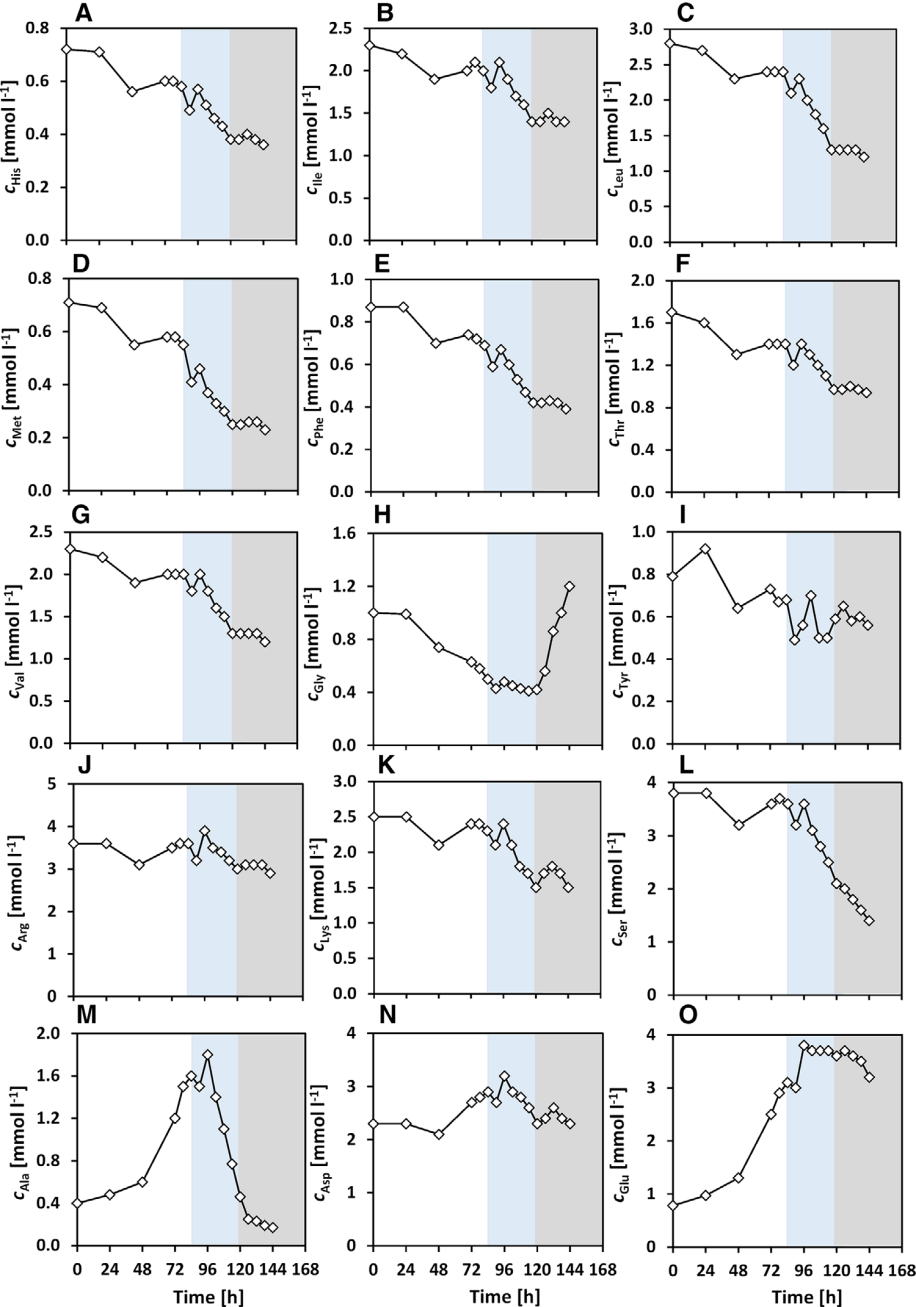
Amino acid profiles from batch cultivation with metabolic shift to lactate consumption, single measurements. Phases indicated: lactate formation phase (white), lactate consumption phase (blue) and death phase (grey)

**FIGURE 5:**
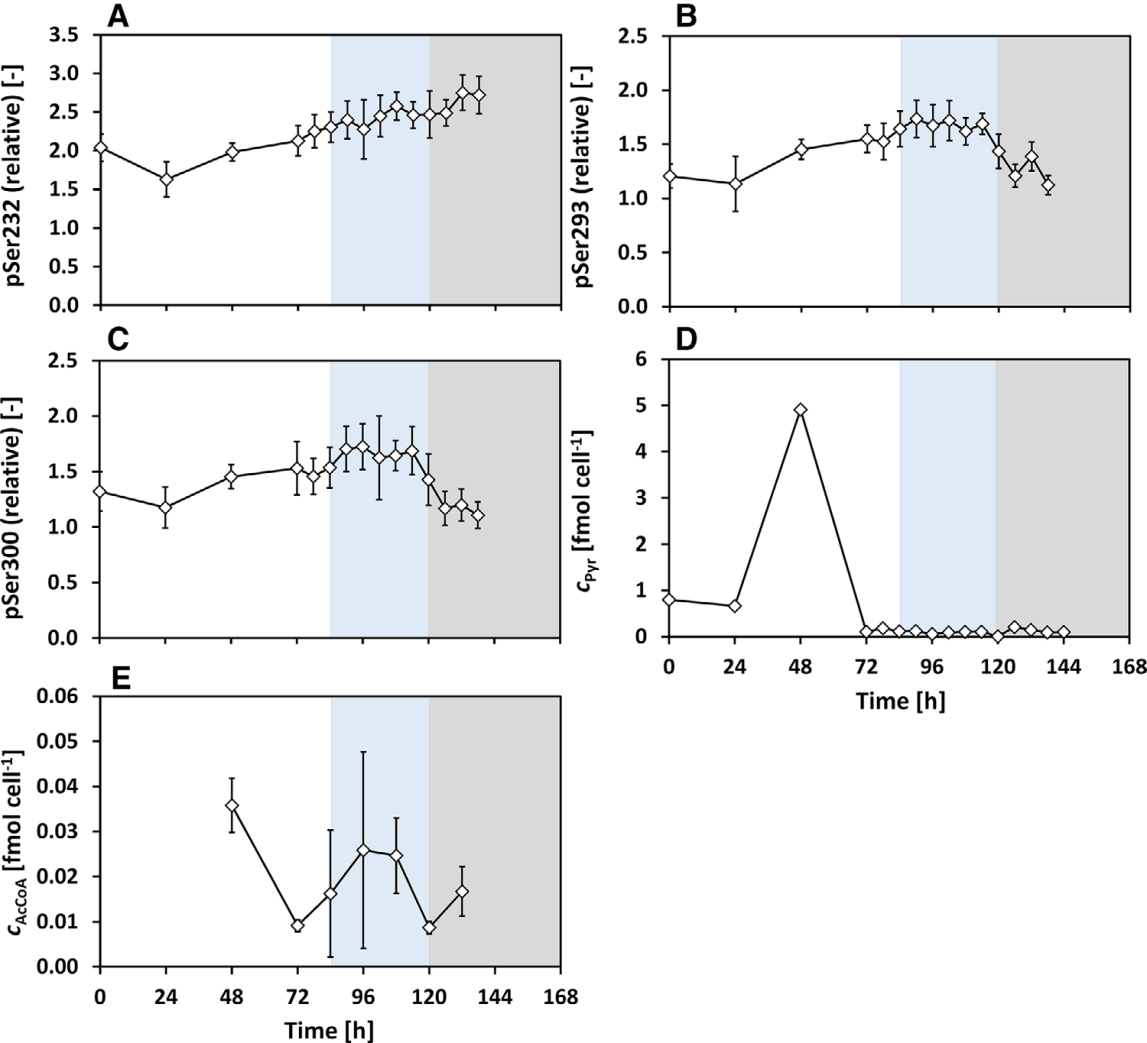
PDC E1α phosphorylation ($P=\frac{\tilde{I_{\mathrm{pSer},E1\alpha }}}{\tilde{I_{E1\alpha }}}$, pSer (relative) = $\frac{P}{P_{0}}$), intracellular pyruvate and acetyl‐CoA levels during batch cultivation with metabolic shift to lactate consumption. Phases indicated: lactate formation phase (white), lactate consumption phase (blue) and death phase (grey). Error bars are standard deviations of technical triplicates for pSer232 and pSer300, technical duplicates for pSer293, pyruvate and acetyl‐CoA

In this study, the PDCE1α phosphorylation is investigated during the metabolic switch from lactate formation to lactate uptake. Antibody‐producing CHO cells were cultivated in pH‐controlled bioreactor experiments and frequent sampling was carried out during lactate switch. In the beginning, the changes in the medium concentrations, LDH and apoptotic fractions are shown, followed by the amino acid profiles. Then, the PDCE1α phosphorylation levels and the intracellular pyruvate ($c_{\mathrm{Pyr},i}$) and acetyl‐CoA concentrations ($c_{\mathrm{ACoA},i}$) are presented, followed by a separated discussion of their role in lactate switch. Based on the cell growth and lactate profile (Figure 2A and B), three phases are shown in all figures (Figure 2, 3, 4, 5) and are considered for discussion: lactate formation phase (t=0−84h, white), lactate consumption phase (t=84−120h, blue) and death phase (t=120−144h, grey).

Experimental settings were chosen to avoid two commonly overlaying effects. First, due to the pH control, constant lactate export was ensured [13, 41]. Hence, any effects of pH on cell metabolism were absent. Second, many studies report lactate consumption to occur close to or during the stationary phase, which is usually influenced by cell death mechanisms [42, 43, 44, 45]. Therefore, the initial glucose concentration in the medium was reduced to $20\;\mathrm{mmol}\;L^{-1}$ (typically $42\;\mathrm{mmol}\;L^{-1}$) to maintain steady cell growth and lactate uptake. Please see Supporting Information Subsection 2.1 for detailed information about the pre‐experiments for the development of the experimental design.

### 3.1 Cell growth, apoptosis and LDH activity

As can be seen in Figure 2, cells grew up to a maximum concentration of $X_{v}=10.6\cdot 10^{6}\;\mathrm{cells}\;{\mathrm{mL}}^{-1}$ at t=120h (Figure 2A) with a viability above 96% (not shown). The apoptotic fraction (Figure 2C) was below 5% during both lactate formation and consumption phases. From t=120h onwards, a strong decrease in the viable cell density ($X_{v}=6.8\cdot 10^{6}\;\mathrm{cells}\;{\mathrm{mL}}^{-1}$ at t=144h) was observed. Simultaneously, the apoptotic fraction had increased to 45%. Additionally, LDH activity (Figure 2D) was quantified in the medium as a marker of cell lysis [46, 47]. During lactate formation phase and lactate consumption, LDH activity was low and upon the onset of death phase after t=120h, it increased by nearly 10 times to 270 $U\;L^{-1}$. This increase in LDH activity indicates that the cells undergo lytic cell death in addition to apoptosis.

### 3.2 Metabolites and metabolic rates

The changes of the metabolites are shown in Figure 2. It should be noticed, that specific rates for glucose uptake and lactate formation and lactate uptake were calculated based on the number of cells (i.e., $X_{v}$), as described in Subsection 2.7.3. To evaluate changes in the cell volume, the median cell diameter is shown in Figure 3A. During the exponential phase, the median cell diameter was 12.78±0.14μm (average ± standard deviation) and it decreased only by a negligible extent to 12.40±0.17μm during lactate consumption.

#### 3.2.1 Glucose and lactate

The glucose concentration (Figure 2E) had an initial value of $c_{\mathrm{Glc}}=18.6\;\mathrm{mmol}\;L^{-1}$ and glucose was fully depleted at t=84h. By this time, the lactate concentration reached a maximum of $c_{\mathrm{Lac}}=29.6\;\mathrm{mmol}\;L^{-1}$ (Figure 2B) and the cells started to take up lactate immediately thereafter. Lactate uptake stopped at $c_{\mathrm{Lac}}=9.6\;\mathrm{mmol}\;L^{-1}$ (t=126h), without any further lactate depletion. After t=120h, cell death (Figure 2A) began and the subsequent slower decrease in extracellular lactate concentration was possibly a combination of reduced cell metabolic activity and increasing LDH activity in the medium. Thus, lactate was taken up by the cells immediately after glucose depletion, with no significant adaption time or increased cell death and the cell viability was maintained during the lactate consumption phase.

#### 3.2.2 Glucose uptake rate

The cell‐specific glucose consumption rate ($q_{\mathrm{Glc}}$, Figure 3B) was the highest during exponential growth with $q_{\mathrm{Glc},\max}=0.14\;\mathrm{pmol}\;{\mathrm{cell}}^{-1}\;h^{-1}$ between t=24h−48h. During low glucose conditions ($c_{\mathrm{Glc}}<\;5\;\mathrm{mmol}\;L^{-1}$) between t=72h−84h, $q_{\mathrm{Glc}}$ was drastically reduced and was $\approx 0\;\mathrm{pmol}\;{\mathrm{cell}}^{-1}\;h^{-1}$ upon glucose depletion at t=84h.

#### 3.2.3 Lactate formation and uptake rate

The cell‐specific lactate formation rate ($q_{\mathrm{Lac}}$, Figure 3C) shows a similar trend compared to $q_{\mathrm{Glc}}$ up to t=84h, with $q_{\mathrm{Lac},\max}=0.25\;\mathrm{pmol}\;{\mathrm{cell}}^{-1}\;h^{-1}$. After glucose depletion, lactate consumption began with $q_{\mathrm{Lac}}=-0.07\;\mathrm{pmol}\;{\mathrm{cell}}^{-1}\;h^{-1}$, which is almost 3.6‐fold lower than the absolute value of specific lactate formation rate. The specific consumption rate declined steadily to $q_{\mathrm{Lac}}=-0.04\;\mathrm{pmol}\;{\mathrm{cell}}^{-1}\;h^{-1}$ right before cell death began.

#### 3.2.4 Glutamine and ammonia

The initial concentration of glutamine was $c_{\mathrm{Gln}}=6\;\mathrm{mmol}\;L^{-1}$, which was depleted at t=84h (Figure 2F), comparable to glucose. Ammonium was formed constantly during the entire duration of the cultivation and reached a maximum concentration of $c_{\mathrm{Amm}}=8.5\;\mathrm{mmol}\;L^{-1}$ (Figure 2G) at t=144h.

#### 3.2.5 Antibody

The peak antibody titer was $c_{\mathrm{Ab}}=85\;\mathrm{mg}\;L^{-1}$ at the end of the cultivation, as seen in Figure 2H. The product concentrations were comparable to previous studies using the same medium and cell line [43, 48, 49, 50].

#### 3.2.6 Oxygen uptake rate

Between t=0h−37h, no additional oxygen supply was needed and the increasing $X_{v}$ resulted in a decreasing $q_{\mathrm{OUR}}$ (Figure 3D, Subsection 2.7.4). From t=37h onwards, the dissolved oxygen decreased to 40% and was subsequently maintained at this level by oxygen sparging. A peak value of $q_{\mathrm{OUR}}=5.7\;\mathrm{pmol}\;{\mathrm{cell}}^{-1}\;h^{-1}$ is seen at t=54h, which then declined steadily. This trend continued even after glucose and glutamine depletion and the switch to lactate consumption at t=84h. $q_{\mathrm{OUR}}$ decreased steadily with further substrate depletion and toward the end of the cultivation, $q_{\mathrm{OUR}}$ was $1.7\;\mathrm{pmol}\;{\mathrm{cell}}^{-1}\;h^{-1}$. The gas flows and offgas composition can be seen in Supporting Information Figure 5.

### 3.3 Amino acids

The amino acids which are generally classified to be essential for CHO cultures (Figure 4A–G), such as histidine ($c_{\mathrm{His}}$), isoleucine ($c_{\mathrm{Ile}}$), leucine ($c_{\mathrm{Leu}}$), methionine ($c_{\mathrm{Met}}$), phenylalanine ($c_{\mathrm{Phe}}$), threonine ($c_{\mathrm{Thr}}$) and valine ($c_{\mathrm{Val}}$) were constantly consumed during the entire cultivation, but were not depleted [51, 52]. Only small differences in their uptake can be seen between the lactate formation and consumption phases. Glycine ($c_{\mathrm{Gly}}$) was consumed only during the lactate formation phase, and the concentration changes were rather small during lactate consumption (Figure 4H). Then, $c_{\mathrm{Gly}}$ increased during cell death. The tyrosine concentration ($c_{\mathrm{Tyr}}$, Figure 4I) did not change much and an average value of $c_{\mathrm{Tyr}}=0.6\;\mathrm{mmol}\;L^{-1}$ was maintained. Arginine ($c_{\mathrm{Arg}}$), lysine ($c_{\mathrm{Lys}}$) and serine ($c_{\mathrm{Ser}}$) showed very small changes in concentration during the lactate formation phase and are seen to be consumed once lactate consumption begins (Figure 4J–L). Alanine ($c_{\mathrm{Ala}}$), aspartate ($c_{\mathrm{Asp}}$) and glutamate ($c_{\mathrm{Glt}}$) were produced during lactate formation phase (Figure 4M–O). Only alanine and aspartate are seen to be consumed during lactate consumption, while glutamate levels remain constant. The switch to alanine uptake upon glucose or glutamine limitation has been previously reported [51, 53]. Alanine has been suggested to serve as a carbon source for the TCA cycle through transamination‐derived pyruvate when glucose is scarce [45, 54]. Aspartate, which is an important product of glutamine anaplerosis, plays an important role in nucleotide synthesis and can contribute to the electron transfer chain through the malate–aspartate shuttle. It has been known to play a crucial role in a glutamine‐limited environment [55]. Overall, the metabolic switch to lactate consumption did not depend on amino acid depletion (except glutamine), which is also supported by the constant formation of ammonia through amino acid consumption (Figure 2G) [17, 45].

### 3.4 Regulation of PDC E1α

The PDC E1α phosphorylation levels were quantified by indirect flow cytometry (Subsection 2.8) at the sites Ser232, Ser293 and Ser300. To account for variations in the expression of PDC E1α (see Supporting Information Figure 6), the PDC phosphorylation levels were normalized on the PDC E1α amount (see Subsection 2.8). The changes in the relative phosphorylation levels of the three residues are shown in Figure 5A–C. The corresponding changes in the intracellular levels of pyruvate and acetyl‐CoA are shown in Figure 5D and E, respectively.

#### 3.4.1 PDC E1α phosphorylation

The relative phosphorylation of Ser232 (Figure 5A) increased throughout the culture duration, from a minimum of 1.63 at t=24h to a maximum value of 2.72 (66% increase, calculated on minimum value). The changes in pSer293 and pSer300 show similar trends, which are different from the pSer232 changes. As can be seen in Figure 5B and C, pSer293 and pSer300 increased slightly starting at 1.2 at the beginning of the culture to 1.6 (33% increase, calculated on minimum) at the end of the lactate formation phase (t=84h). After the switch to lactate consumption, the phosphorylation state was constant, varying slightly between 1.6 ‐ 1.7. At t=114h, the levels of pSer293 and pSer300 began to simultaneously decrease until ≈1.2 at t=126h. A higher phosphorylation level indicates a deactivation of PDC and vice versa [34]. Here, it can be inferred that PDC became increasingly inactive in the exponential phase and that PDC was more inactive during the early lactate consumption phase.

#### 3.4.2 Pyruvate and acetyl‐CoA

The concentrations of the intracellular metabolites pyruvate and acetyl‐CoA are shown in Figure 5D and E (see Subsection 2.7.2). Starting from an initial pyruvate concentration of $c_{\mathrm{Pyr}}=0.8\;\mathrm{fmol}\;{\mathrm{cell}}^{-1}$ (t=0h−24h), an accumulation of $c_{\mathrm{Pyr}}$ up to $4.9\;\mathrm{fmol}\;{\mathrm{cell}}^{-1}$ is observed at t=48h. Then, the pyruvate concentration decreased below the detection limit and was consistently low. This prominent spike was confirmed in two independent cultivations (second cultivation shown in Supporting Information Figure 7). Acetyl‐CoA concentrations (Figure 5E) were roughly two orders of magnitude lower compared to that of pyruvate, indicating a negligible amount. During the exponential phase at t=48h, the acetyl‐CoA level was the highest, at about $c_{\mathrm{AcCoA}}=0.036\;\mathrm{fmol}\;{\mathrm{cell}}^{-1}$ after which it decreased and was at an average concentration of $c_{\mathrm{AcCoA}}=0.017\;\mathrm{fmol}\;{\mathrm{cell}}^{-1}$.

### 3.5 Discussion

#### 3.5.1 Lactate switch from a physiological perspective

The cells switched to lactate consumption without showing any signs of increased cell death in form of apoptosis or cell lysis (Figure 2). Since other factors such as temperature, dissolved oxygen and amino acids were sufficient for supporting aerobic cell growth, the metabolic shift to lactate consumption was influenced primarily by the depletion of glucose and glutamine. The cells consume more oxygen during exponential growth than during lactate consumption (Figure 3D). [15] suggested that although most of the energy during lactate consumption comes from the TCA cycle, the glucose‐based growth phase is marked by oxidative phosphorylation of NADH and ${\mathrm{FADH}}_{2}$ produced rapidly outside the TCA cycle [15]. Such a segregated metabolism would explain our observations of higher oxygen consumption during the exponential growth phase with lactate formation. Although the cells consume lactate without increased cell death, it was not completely consumed. This could be attributed to signal factors [42, 48] or degradation of growth factors, which were not targeted in this work.

Since glucose and glutamine were depleted at the same time during lactate consumption, the cells switched to take up alanine, aspartate, and other amino acids to maintain biosynthesis and proliferation. This effect is also seen in the steady increase of ammonia after glutamine depletion. The antibody formation was not found to increase during lactate consumption, even though the uptake of amino acids continued. In contrast to fed‐batch cultures with improved productivities [12, 29], cell growth continued in this study during lactate consumption for the ensuing 36 h (Figure 2), which could suggest that CHO DP‐12 cells divert metabolic intermediates toward cell growth.

#### 3.5.2 Role of PDC regulation

The PDCE1α phosphorylations quantified in this work indicate that PDC is increasingly deactivated during exponential growth (up to t=72h, Figure 5), as also presumed in literature [13]. During lactate consumption, the phosphorylation levels of the serine residues were expected to be lower to allow pyruvate to enter the TCA cycle due to increased oxidative metabolism [12, 28, 29]. Here, during early lactate consumption (t=90h−114h) the phosphorylation levels of Ser293 and Ser300 were rather constant, at a 33% higher level when compared to glucose excess phase. Such an increase in PDC phosphorylation and steady cell growth and productivity at the same time could hint that pyruvate is metabolized in alternative pathways (e.g., phosphoenolpyruvate as phosphate donor), as observed in primary cancer cells [56, 57].

Phosphorylation of Ser232 is seen to behave very differently compared to Ser293 and Ser300, with the phosphorylation level increasing even during lactate consumption. [36] used a molecular dynamics method and could not identify inhibitory effects due to pSer232 in the cases where pSer293 and pSer300 inhibited enzyme activity [36]. Although in vitro studies have shown that the phosphorylation of any of the three Ser residues on the E1α subunit of PDC leads to the deactivation of the enzyme complex [35], pSer293 was found to have the most potent inhibitory effect [58, 59]. Moreover, [60] suggest that phosphorylation at Ser293 is the most dominating effect in vivo [60]. The here observed increased phosphorylation of Ser232 might have different effects than the conventionally assumed deactivation of PDC.

#### 3.5.3 Role of pyruvate in lactate metabolism

Pyruvate is a key metabolite that represents an intermediate in cell metabolism at which the cells mainly divert glucose carbon either toward lactate or toward oxidative phosphorylation [19, 61]. PDC catalyzes the conversion of pyruvate to acetyl‐CoA, allowing the glucose‐derived pyruvate to enter the TCA cycle. The intracellular pyruvate levels observed in this work indicate that pyruvate strongly accumulates during the exponential growth phase with lactate formation. Generally, high rates of aerobic glycolysis could lead to an increased availability of pyruvate in the cytosol, allowing the formation of lactate and also alanine [61]. In our study, pyruvate was observed to accumulate in the cells during lactate formation and was very low during lactate uptake, indicating the same phenomena as described by [61]. The observed pyruvate excess is supported by the concurrent excretion of alanine (Figure 4M) during aerobic glycolysis, since alanine is formed primarily through the transamination reaction between pyruvate and glutamate [29, 62, 63]. Due to the reversible exchange of pyruvate across the cell membrane, it is likely that the extracellular pyruvate level follows a similar trend (not measured) [41, 64, 65]. During lactate uptake, as can be seen in Figure 3C, the absolute value of $q_{\mathrm{Lac}}$ is comparatively lower than that during lactate formation, and the observed lack of pyruvate accumulation during lactate uptake indicates that the activity of PDC is high enough to metabolize the pyruvate formed from lactate or the pyruvate is metabolized by alternative pathways as previously stated. This indicates a reduced pyruvate flux into the TCA cycle, which is also confirmed by the reduced $q_{\mathrm{OUR}}$ (Figure 3D) during lactate uptake. Acetyl‐CoA, on the other hand, shows almost no accumulation. High acetyl‐CoA levels are also known to reduce PDC activity [34]. This effect was found to be negligible in this work since very low levels of acetyl‐CoA were detected.

## 4 CONCLUDING REMARKS

For the first time, the interaction of oxidative metabolism with PDC phosphorylations is investigated in this study during the metabolic shift to lactate consumption in antibody‐producing CHO cells. Contrary to the general assumption of reduced phosphorylation levels during lactate uptake (i.e., TCA up‐regulation), the relative phosphorylation of all three phosphorylation sites was higher during lactate consumption compared to the exponential growth phase. The relative phosphorylation levels of Ser293 and Ser300 were found to be constant during the initial phase of lactate consumption and to decline in the late lactate consumption phase. The phosphorylation of Ser232 increased steadily. These findings suggest that the oxidative metabolism was reduced in CHO DP‐12 cells during lactate uptake, also confirmed by the decreasing $q_{\mathrm{OUR}}$. The intracellular pyruvate concentration was found to rise during lactate formation and was very low during lactate consumption, and acetyl‐CoA showed nearly no accumulation. It is clear that pyruvate metabolism and PDC regulation show characteristic changes during lactate switch. However, the phosphorylation of PDC, one of the important regulation mechanisms, alone cannot completely explain the experimental observations. Alternative pathway(s) and the regulation of pyruvate metabolism may be involved. It can be stated that PDC regulation is much more complex than so far understood, even in the field of well established and characterized CHO processes. To our knowledge, this is the first study which provides data on PDC regulation by phosphorylation under dynamic process conditions. This knowledge could help gauge the importance of PDC and pyruvate better when designing genetic engineering or feeding strategies to regulate lactate metabolism. Further experimental and model‐based studies are needed to enable a more systematic and quantitative understanding of the dynamic regulations of pyruvate metabolism and PDC, which have important implications for both mammalian cell culture and human diseases.

## NOMENCLATURE

**Table:** 

Variable	Explanation	Unit
$c_{j,i}$	Concentration of substance j at time i	[$\mathrm{mmol}\;L^{-1}$]
$q_{\mathrm{Glc}}$	Cell‐specific glucose uptake rate	[$\mathrm{mmol}\;{\mathrm{cell}}^{-1}\;h^{-1}$]
$q_{\mathrm{Lac}}$	Cell‐specific lactate formation/uptake rate	[$\mathrm{mmol}\;{\mathrm{cell}}^{-1}\;h^{-1}$]
$q_{\mathrm{OUR}}$	Cell‐specific oxygen uptake rate	[$\mathrm{pmol}\;{\mathrm{cell}}^{-1}\;h^{-1}$]
$X_{v}$	Viable cell density	[$\mathrm{cells}\;{\mathrm{mL}}^{-1}$]

## ABBREVIATIONS

**Table:** 

Abbreviation	Explanation
Ab	antibody
ACoA	acetyl‐CoA
Ala	alanine
Amm	ammonium
Arg	arginine
Asp	aspartate
CHO	Chinese hamster ovary
DAPI	4',6‐diamidino‐2‐phenylindole
FSC	forward scatter
Glc	glucose
Gln	glutamine
Glt	glutamate
Gly	glycine
His	histidine
Ile	isoleucine
Lac	lactate
LDH	lactate dehydrogenase
Leu	leucine
Lys	lysine
Met	methionine
PBS	phosphate buffered saline
PDC	pyruvate dehydrogenase complex
PDK	pyruvate dehydrogenase kinase
PDP	pyruvate dehydrogenase phosphate phosphatase
Phe	phenylalanine
pSer	phosphorylated serine
Pyr	pyruvate
Ser	serine
SSC	side scatter
TCA	tricarboxylic acid cycle
Thr	threonine
Tyr	tyrosine
Val	valine

## Supporting information

Supporting Information
