# Supplementary material for: Regulation of pyruvate dehydrogenase complex related to lactate switch in CHO cells
Source: Eng Life Sci. 2020 Sep 28;21(3-4):100–14. doi: 10.1002/elsc.202000037 (PMC7923601; doi:10.1002/elsc.202000037)
Supplement: Supplementary file 1 — Supporting Information [file ELSC-21-100-s001.pdf]

Supporting Information to: Regulation of pyruvate dehydrogenase complex related to lactate switch in CHO cells

Johannes Möller*†, Krathika Bhat*, Lotta Guhl, Ralf Pörtner, Uwe Jandt, An-Ping Zeng‡

August 4, 2020

1 Supporting Material and Methods

1.1 Flow cytometry gating for apoptosis measurements

Annexin V/PI staining was used to track the increase in the apoptotic cell population during cultivations. First, the single cells were gated by SSC-A vs. FSC-A scatter plot of all events, and FSC-H vs. FSC-A scatter plot, as shown in the Supporting Information to [1]. In order to classify the populations seen in annexin V/PI stained cells, the gating strategy is shown in Supporting Figure 1. The density scatter plot of PB450-A vs. PC5.5-A for

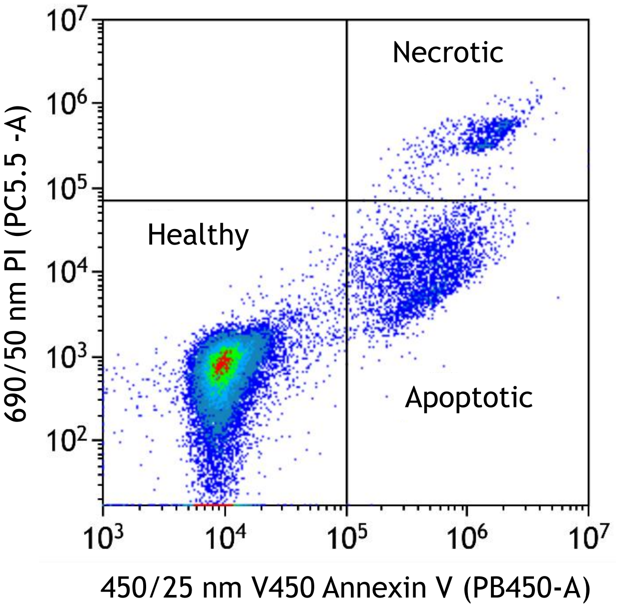

Supporting Figure 1: Gating for annexin V/PI staining applied to exponentially growing CHO DP-12 cells (48 h)

the living cell fraction can be divided into three regions. The lower left regions represent the healthy cells which are negative for both annexin V and PI. The lower right region represents apoptotic cells, which are positive only for annexin V, indicating no loss of membrane integrity. The upper right region represents necrotic and late apoptotic cells with compromised membranes and are hence positive for both annexin V and PI [2, 3].

1.2 Indirect flow cytometry assay for analysis of PDC phosphorylation

Principle The basis of the indirect flow cytometry assays is the binding of phosphorylation-specific non-conjugated antibodies (i.e., primary staining) at the individual PDC E1α-regulating sites. Then, fluorophore-conjugated antibodies are added, they bind to the primary antibodies, and the fluorescence signals are measured with flow cytometry. The signal intensity thereby correlates with the amount of bound pSer antibodies and reflects the PDC E1α phosphorylation. As can be seen in Supporting Figure 2, the cell membrane is permeabilized at the beginning to enable antibody entry. Then, unspecific binding sites are blocked using a blocking solution containing glycine and fetal calf serum (FCS), and the individual PDC E1α phosphorylations are labeled using pSer-specific antibodies. In the last step, the primary pSer-bound antibodies and PDC E1α itself are labeled with fluorophore-conjugated antibodies, and the PDC E1α phosphorylation is quantified using flow cytometry. The individual steps are described in the following.

Cell fixation The cells were sampled from the bioreactor, and the cell suspension was centrifuged ($1000 \cdot g$, 3 min), and the supernatant was discarded. Cells were suspended in 4% Paraformaldehyde (HistoFix, Roth) and incubated at room temperature for 20 min. Afterward, the fixed cells were centrifuged ($400 \cdot g$, 10 min) and washed with PBS. This was repeated three times, and the cells were stored in the fridge (4°C).

Buffers The used buffers and their composition are listed in Table 1. All buffers were centrifuged at $10000 \cdot g$ for 10 min and subsequently filtered (0.22 µm, Roth).

Reagents The used reagents are listed in Table 2.

Assay steps First, the number of cells was determined in the fixed cell solution using flow cytometry (Cytoflex, Beckman Coulter), and the fixed cell suspension was diluted with PBS to achieve $0.5 \cdot 10^6$ cells ml^{-1}. Out of this

*Authors contributed equally.
†Johannes Möller and An-Ping Zeng (corresponding authors); Hamburg University of Technology, Bioprocess and Biosystems Engineering, Denickestr. 15, 21073 Hamburg, Germany, e-mail: `johannes.moeller@tuhh.de`,
‡e-mail: `aze@tuhh.de`

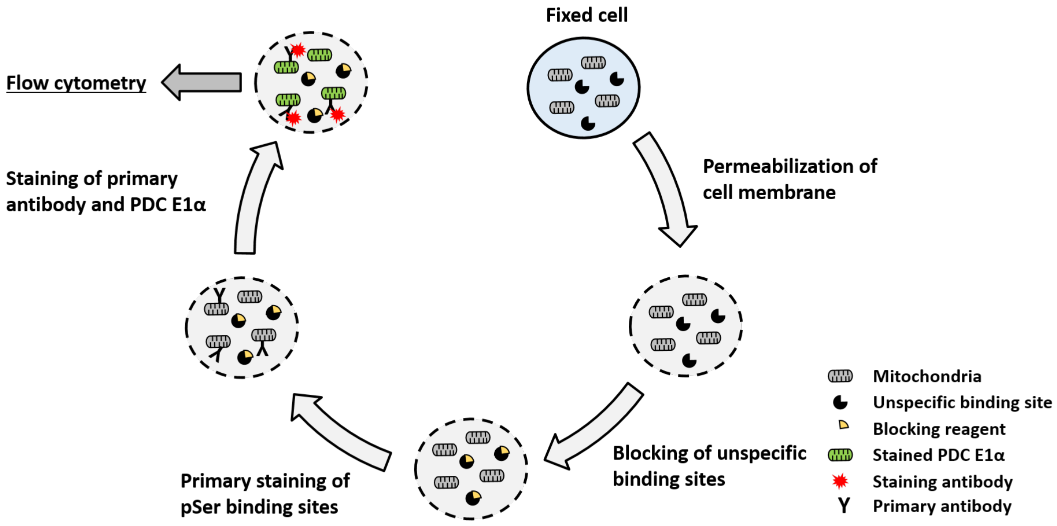

Supporting Figure 2: Schematic workflow for indirect flow cytometry assay to determine the relative PDC phosphorylation, notice that each pSer PDC E1α phosphorylation needs to be determined separately.

Supporting Table 1: Buffers for indirect flow cytometry assay to quantify PDC E1α phosphorylation. Between all steps, intermediate washing was performed.

Buffer	Composition [% (v/v)]
Permeabilization	2% Tween 20 (Sigma-Aldrich)
	98% PBS (Sigma-Aldrich)
Blocking	10% blocking solution (Abcam, In-Cell ELISA support pack)
	10% fetal calf serum (FCS) (Biochrom, Germany)
	80% 0.33 mol l^{-1} Glycine (Sigma-Aldrich) in permeabilization buffer
Staining	10% blocking solution (Abcam, In-Cell ELISA support pack)
	10% FCS
	80% PBS
Washing	0.25% Tween 20 (400x, Abcam, In-Cell ELISA support pack)
	99.75% PBS

cell suspension, 200 µl were transferred to each well of a 96-well plate (v-shaped, Thermo Scientific, Germany). In total, 14 wells were filled per sample, from which six were without primary antibody and three wells for each PDC E1α antibody (pSer232, pSer293, pSer300, respectively). The plate was then centrifuged (Hettich Universal 320 R, Hettich, Germany) at 400 g for 15 min and 150 µl supernatant was taken out and replaced with washing buffer and the cell pellet was resuspended (Biomek 4000, Beckman Coulter). These steps were repeated twice (centrifugation time reduced to 10 min) and they together constitute the washing sequence. In the last step, the cell pellet was manually suspended in permeabilization buffer. The plate was incubated (400 rpm, 20 min, $20°$C, Thermomixer, Eppendorf, Germany) and a washing sequence was performed. At the last step, the cells were resuspended manually in the blocking buffer and further incubated (400 rpm, 2 h, $20°$C, Thermomixer, Eppendorf).

After a washing sequence, 0.5 µl anti-PDC E1α subunit antibody solution containing either no anti-PDC E1α pSer antibody or just one anti-PDC E1α pSer antibody solution was added in 150 µl staining solution to each well. The plate was sealed and incubated overnight (400 rpm, 16 h, $4°$C, Thermomixer). Afterward, the plate was washed and 150 µl staining buffer including 0.05 µl anti-rabbit antibody was added to each well and further incubated (400 rpm, 20 min, $20°$C, Thermomixer). The samples were measured by flow cytometry (Cytoflex, Beckman Coulter) after the final washing sequence.

Flow cytometry Gating was performed to distinguish the fixed cells (Supporting Figure 3 a) first, and the single cells were determined (Fig. 3 b). Then, PDC E1α positive cells were gated in the 525/40 nm filter (Fig. 3 c) between the intensity of $\approx 10^4 - 10^6$. From these cells, the pSer E1α positive gate was derived in the 450/45 nm filter (Fig. 3 d).

Out of the flow cytometer data, the median of the individual gated positive intensities (E1α positive-$\widetilde{I_{\mathrm{E1}\alpha}}$ and pSer E1α positive-$\widetilde{I_{\mathrm{pSer,E1}\alpha}}$, respectively) were determined and the relative phosphorylation P was quantified:

$$P = \frac{\widetilde{I_{\mathrm{pSer,E1}\alpha}}}{\widetilde{I_{\mathrm{E1}\alpha}}}. \qquad (1)$$

Due to the biological and data-based fluctuations, P was standardized on the non-primary labeled intensity P_0 (i.e., no pSer antibody) measurements, and pSer (relative) $= \frac{P}{P_0}$ was used for data analysis.

Supporting Table 2: Reagents for indirect flow cytometry assay to quantify PDC E1α phosphorylation.

Reagent	Concentration
Anti-PDC E1α pSer232 (polyclonal-rabbit, Merck, Germany)	1 µg ml^{-1}
Anti-PDC E1α pSer293 (polyclonal-rabbit, Merck, Germany)	1 µg ml^{-1}
Anti-PDC E1α pSer300 (polyclonal-rabbit, Merck, Germany)	1 µg ml^{-1}
Anti-PDC E1α (monoclonal-mouse, G-biosciences, USA, Alexa-fluor 488)	1.25 µg ml^{-1}
Anti-rabbit (polyclonal-goat, Abcam, USA, Alexa-fluor 405)	0.25 µg ml^{-1}

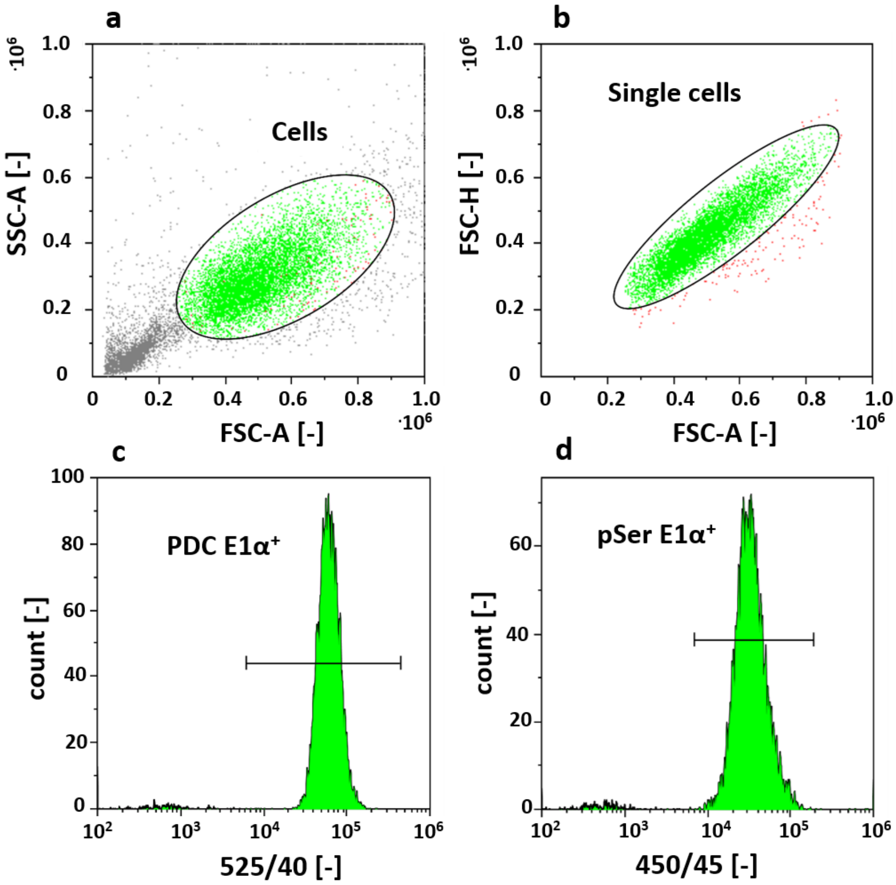

Supporting Figure 3: Flow cytometry gating strategy used in the quantification of PDC E1α phosphorylation. A: SSC-A vs. FSC-A with gating for cells; B: FSC-H vs. FSC-A with gating for single cells; C: PDC E1α positive gate; D: pSer positive gate; n=10000 events.

1.3 Influence of initial glucose concentration on lactate consumption

CHO DP-12 cells were cultivated in shake flasks (40 ml working volume, Corning) in medium with reduced glucose concentrations between 5 mmol l^{-1} – 30 mmol l^{-1} (6 mmol l^{-1} glutamine, 200 nmol l^{-1} methotrexate). This range was used due to the previously identified absence of lactate uptake between 32.5 mmol l^{-1} – 52.5 mmol l^{-1} [1, 4]. Therefore, a stock solution of medium with 1 mol l^{-1} glucose was prepared by adding glucose (Sigma-Aldrich, Germany) to the medium without glucose (Xell AG). Five different initial glucose concentrations (5, 10, 15, 20, and 30 mmol l^{-1}) were tested, and the medium was prepared by diluting the stock medium as required. The incubator conditions were as explained in the main manuscript, and the initial cell density was $0.3 \cdot 10^6$ cells ml^{-1}.

2 Supporting Results and Discussion

2.1 Pre-experiments

The investigation of dynamic PDC E1α phosphorylations during the lactate switch requires a reproducible experimental setting for the triggering of the metabolic change from lactate formation to lactate uptake. So far, certain observations were made with respect to lactate uptake in CHO DP-12 cells in previously published studies even though the lactate metabolism was not specifically targeted:

1. maximal lactate concentration of $c_{\text{Lac}} \approx$ 20 mmol l^{-1} was determined in batch and fed-batch pH uncontrolled shake flask cultures, regardless of the initial glucose concentration [1, 4]
2. lactate uptake began with glucose depletion in fed-batch bioreactor cultures [5]
3. cell death and lactate uptake took place at the same time [5]

Here, the influence of the initial concentration of glucose was investigated in shake flask cultures below the standard concentration of 42 mmol l^{-1} (Subsection 2.2 in main manuscript) with the aim of reproducibly inducing lactate uptake. The effect of the initial glutamine concentration was tested in a previous experiment, and no effect was found on lactate uptake in shake flask cultures under the given conditions (not shown). At first, CHO cells were cultivated in medium with reduced glucose concentrations between 5 mmol l^{-1}–30 mmol l^{-1} (6 mmol l^{-1} glutamine). As can be seen in Supporting Figure 4 a, cell growth was comparable for 30 mmol l^{-1} and 20 mmol l^{-1} initial glucose with up to 10 $\cdot$ 10^6 cells ml^{-1} at 144 h. Reduced growth ($\approx 7 \cdot 10^6$ cells ml^{-1} after 144 h) was observed for 15 mmol l^{-1} and 10 mmol l^{-1} initial glucose and cell death occurred earlier for 5 mmol l^{-1} initial glucose. The shape of the glucose concentration courses (Supporting Fig. 4 b) were comparable for all tested initial concentrations.
Lactate (Supporting Fig. 4 c) was comparably formed in all tested conditions, and lactate uptake was identified to take place below an initial glucose concentration of 20 mmol l^{-1} in the here tested experimental setting. This reduced initial glucose concentration was subsequently used for the investigation of the lactate switch as targeted in the main manuscript. Only small deviations were observed in the glutamine concentrations (Supporting Fig. 4d), which could be based on deviations in the viable cell density.

In this experimental setting, for cultivations with initial glucose above 20 mmol l^{-1}, cell growth on glucose is prolonged, resulting in the overlaying effects of concomitant substrate consumption discussed above. This would not allow the clear study of PDC dynamics influenced by lactate consumption alone. For concentrations less than 20 mmol l^{-1}, reduced growth was observed. Hence, to exclude overlaying effects of glucose consumption and pH in shake flask cultures while achieving a sufficiently long phase with viable cell growth on lactate, an initial glucose concentration of 20 mmol l^{-1} was chosen for further experiments.

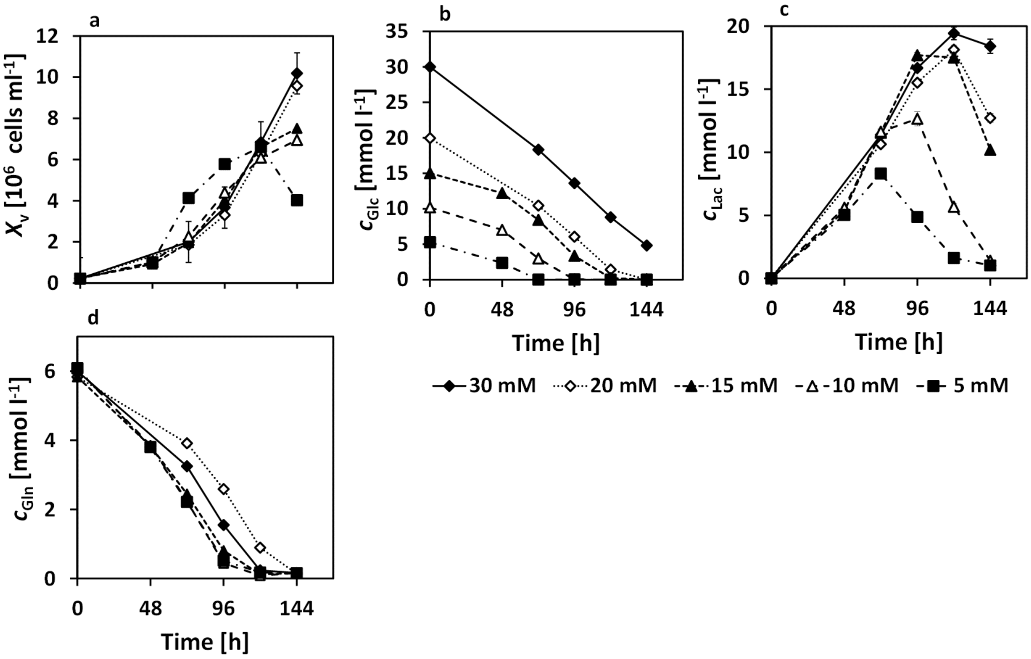

Supporting Figure 4: Comparison of shake flask cultures with varying initial glucose concentrations, error bars and standard deviation of biological one-fold experiments (technical triplicates).

2.2 Gas flow rates

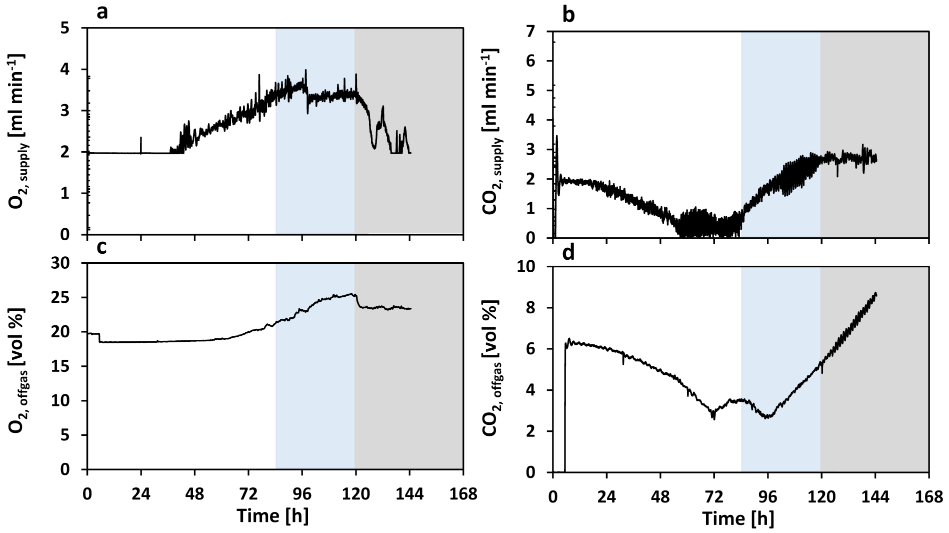

Supporting Figure 5: Gas flow rates and oxygen concentration in offgas of experiment discussed in the main manuscript. The measured data was used to calculate the specific oxygen uptake rate as described in subsection 2.7.2 of the main manuscript. Phases indicated: lactate formation phase (white), lactate consumption phase (blue) and death phase (grey). The gas supply data was obtained from the process control system and the off-gas data was obtained from the gas analyser BlueVary, BlueSens, Germany.

2.3 PDC E1α phosphorylation

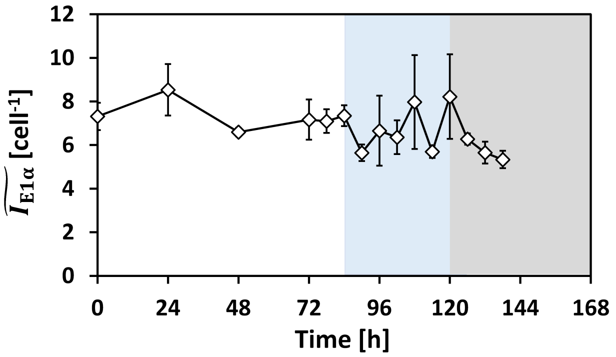

Supporting Figure 6: Mean measured intensity of E1α signal of experiment discussed in the main manuscript. Phases indicated: lactate formation phase (white), lactate consumption phase (blue) and death phase (grey). Average and standard deviation of 14 measurements per time point. Please see Supporting Subsection 1.2 for more information.

2.4 Evaluation of intracellular pyruvate concentration changes

Based on the lactate profile in this experiment, two phases are considered: lactate formation phase ($t = 0 - 90$ h, white) and lactate consumption phase ($t = 90 - 168$ h, blue).

Cell growth The cell growth, as shown in Supporting Figure 7a shows a peak cell count of $X_\mathrm{v} = 6.9 \cdot 10^6$ cells ml^{-1} at $t = 168$ h. The viability was greater than 90% up to $t = 144$ h, after which it declined to 83% and the cultivation was terminated (not shown). The apoptotic fraction in the population showed no drastic increase during lactate consumption and was at an average value of 8% until $t = 120$ h (Fig. 7b).

Glucose and Lactate Glucose was at an initial concentration of $c_\mathrm{Glc} = 17.7$ mmol l^{-1} and was completely depleted at $t = 90$ h (Fig. 7c). The lactate concentration reached a peak value of $c_\mathrm{Lac} = 27$ mmol l^{-1} (Fig. 7d) at $t = 90$ h and there were very small changes in concentration up to $t = 102$ h. The cell-specific lactate consumption rate increased from $t = 102$ h onward and the lactate concentration dropped to $c_\mathrm{Lac} = 5$ mmol l^{-1} at the end of the cultivation.

Pyruvate The pyruvate concentration profile is shown in Supporting Figure 7e. During the lactate formation phase, the pyruvate concentration was about $c_\mathrm{Pyr} = 0.51$ fmol cell^{-1} up to $t = 48$ h and then accumulated up to $c_\mathrm{Pyr} = 5.57$ fmol cell^{-1} at $t = 72$ h. Once the glucose was limiting and lactate consumption began, the pyruvate concentration decreased to $c_\mathrm{Pyr} = 1.18$ fmol cell^{-1} at $t = 120$ h.

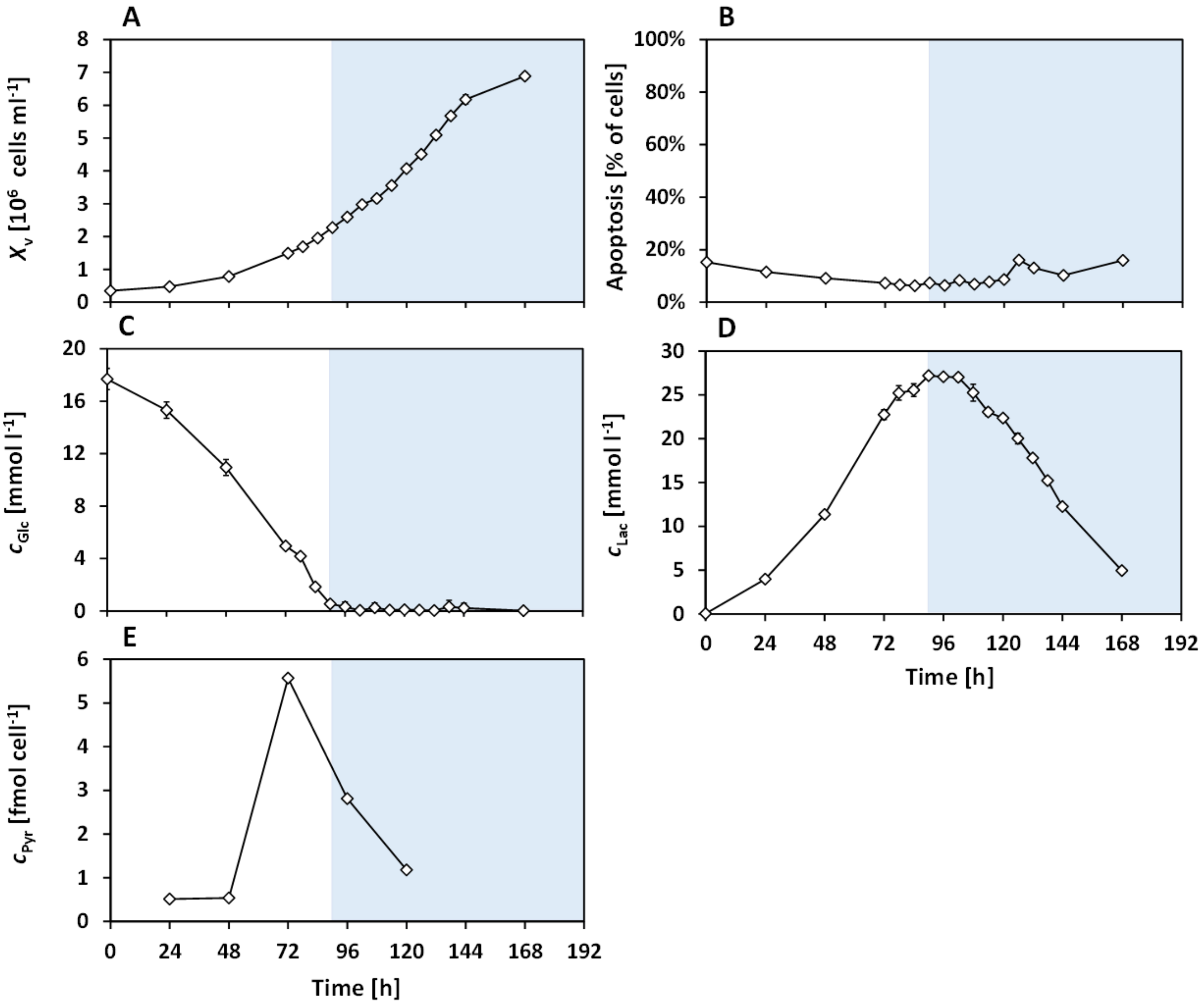

Supporting Figure 7: Mean experimental results from batch cultivation performed as a supporting experiment to evaluate intracellular pyruvate changes (20 mmol l^{-1} initial glucose, 6 mmol l^{-1} initial glutamine, 200 ml initial working volume, $0.3 \cdot 10^6$ cells ml^{-1} initial cell density. Phases indicated: lactate formation phase (white) and lactate consumption phase (blue); error bars are standard deviations of three technical measurements from a single bioreactor run.

3 Nomenclature

Variable	Explanation	Unit
c_i	concentration of component i	[mmol l^{-1}]
$I_{E1\alpha}$	E1α positive intensity	[-]
$I_{pSer,E1\alpha}$	pSer E1α positive intensity	[-]
P	relative phosphorylation	[-]
P_0	standardized relative phosphorylation	[-]
X_v	viable cell density	[cells ml^{-1}]

4 Abbreviations

Abbreviation	Explanation
FCS	fetal calf serum
FSC	foreward scatter
Glc	glucose
Gln	glutamine
Lac	lactate
PBS	phosphate buffered saline
PDC	pyruvate dehydrogenase complex
Pyr	pyruvate
SSC	side scatter

References

[1] J Möller, KB Kuchemüller, T Steinmetz, KS Koopmann, and R Pörtner (2019) "Model-assisted Design of Experiments as a concept for knowledge-based bioprocess development". *Bioprocess Biosyst. Eng.*: DOI: 10.1007/s00449-019-02089-7

[2] D Baskic, S Popovic, P Ristic, and NN Arsenijevic (2006) "Analysis of cycloheximide-induced apoptosis in human leukocytes: Fluorescence microscopy using annexin V/propidium iodide versus acridin orange/ethidium bromide". *Cell Biol. Int.* 30: 924–932. DOI: 10.1016/j.cellbi.2006.06.016

[3] Y Zhang, X Chen, C Gueydan, and J Han (2018) "Plasma membrane changes during programmed cell deaths". *Cell Res.* 28: 9–21. DOI: 10.1038/cr.2017.133

[4] J Möller, T Hernàndez Rodríguez, J Müller, L Arndt, KB Kuchemüller, B Frahm, R Eibl, D Eibl, and R Pörtner (2020) "Model uncertainty-based evaluation of process strategies during scale-up of biopharmaceutical processes". *Comput. Chem. Eng.* 134: 106693. DOI: https://doi.org/10.1016/j.compchemeng.2019.106693

[5] J Möller, K Bhat, K Riecken, R Pörtner, A-P Zeng, and U Jandt (2019) "Process-induced cell cycle oscillations in CHO cultures: Online monitoring and model-based investigation". *Biotechnol. Bioeng.* 116: 2931–2943. DOI: 10.1002/bit.27124
